# Surveillance camera image dataset for visual classification in criminal investigation research (CCTV cameras)

**DOI:** 10.1016/j.dib.2026.113322

**Published:** 2026-10-07

**Authors:** Dimitar Rangelov, Nikolay Rangelov, Radoslav Miltchev, Evgeni Genchev

**Affiliations:** a University of Twente, Drienerlolaan 5, Enschede, 7522 NB, the Netherlands; b Vocational School of Computer Technology, Perusha 4, Pravets, 2161, Bulgaria; c Technical University of Sofia, 8 Kliment Ohridski Blvd., Sofia, 1000, Bulgaria

**Keywords:** Computer vision, Deep learning, Convolutional neural networks, Object recognition, Digital forensics, Law enforcement, Crime scene, Artificial intelligence

## Abstract

This data article describes a curated collection of 1421 images of civilian surveillance and security cameras, assembled to support computer vision research on the visual recognition of camera systems in real-world environments. The images were gathered from freely reusable web sources, open image repositories, and manufacturer product pages, and every image was manually reviewed so that each retained file clearly depicts at least one surveillance camera. The collection covers the most common camera form factors, including dome, pan-tilt-zoom (PTZ), bullet, fixed box, multi-sensor, and consumer IP or doorbell cameras, photographed in outdoor street installations, indoor security setups, pole-mounted and wall-mounted configurations, as well as manufacturer product imagery against plain backgrounds. All files follow a sequential naming convention (camera_<ID>) and are provided in a single directory in JPEG format, in RGB colour space, standardised to a uniform resolution of 640 × 640 pixels. A lightweight annotation file (labels.csv) accompanies the images, recording for each file whether it depicts a camera installed in a real environment (718 in-situ photographs) or a manufacturer-style product image (703 images), together with a per-image camera_type label giving the form factor of the device shown. A documented Python loading script and SHA-256 checksums are distributed with the data. The dataset is publicly available on Zenodo under a CC BY 4.0 licence. It can be reused to train and benchmark image classification and object detection models for automated camera canvassing at crime scenes, and to support research in digital forensics, privacy auditing, urban surveillance mapping, and smart-city applications.

Specifications Table

**Table:** 

Subject	Computer Sciences
Specific subject area	Computer vision and machine learning for visual recognition of surveillance cameras in criminal investigation and digital forensics contexts
Type of data	Image Raw, Table (.csv)
Data collection	Candidate images were located by open-source intelligence (OSINT) searching of freely reusable web sources, open image repositories, and manufacturer product pages; commercial stock-photography sites were excluded. Candidate sources were first validated manually for reuse permissions, images were then retrieved using custom Python scraping scripts, and every retrieved image underwent a second manual validation pass. Each candidate was reviewed manually and through automated checks, and retained only if at least one surveillance camera was clearly visible at adequate quality. Duplicates and low-quality files were excluded. Retained files were renamed sequentially (camera_1 to camera_1421) and consolidated into a single directory. Images were converted to JPEG (RGB) and standardised to 640 × 640 px. No augmentation was applied to the released images. An annotation file (labels.csv) records the context (in_situ or product) and the camera_type (form factor) of each image.
Data source location	The data are hosted on the Zenodo repository (CERN Data Centre, Geneva, Switzerland). Institutions: University of Twente, Enschede, The Netherlands; Technical University of Sofia, Sofia, Bulgaria.
Data accessibility	Repository name: Zenodo Data identification number: 10.5281/zenodo.22669096 Direct URL to data: https://zenodo.org/records/22669096 Instructions for accessing these data: The dataset is openly accessible without registration under a Creative Commons Attribution 4.0 International (CC BY 4.0) licence. The record contains the image archive (combined_dataset.zip), the annotation file (labels.csv), a documented Python loading script (load_dataset.py), a CHECKSUMS.txt file with SHA-256 checksums of the released files, and a README.md, all downloadable directly from the record page. Two DOIs are associated with the deposit and serve different purposes: the concept doi:10.5281/zenodo.19565334 always resolves to the latest version of the dataset and should be used for general citation, whereas the version doi:10.5281/zenodo.22669096 resolves to this specific version and should be used when exact reproducibility is required.
Related research article	None

## 1 Value of the Data

• Surveillance cameras are usually the sensor rather than the subject of image datasets. This collection instead treats the camera itself as the object of interest, providing 1421 openly licensed images of surveillance cameras across six common form factors, varied mounting positions, viewpoints, backgrounds, and lighting conditions. Every image carries both a context label (in-situ or product) and a camera_type label, so the form-factor breakdown can be filtered directly in the released data; a seventh camera_type value, other, holds 33 images in which a camera is present but does not correspond to any of the six form factors.
• Researchers in computer vision can reuse the images and the accompanying labels as positive-class material for training and benchmarking camera/no-camera classifiers. The technical validation reported below makes this concrete: using these images as the positive class, a ResNet-50 classifier reached 98.1% accuracy and a YOLOv8-m classifier 82.0% accuracy on a held-out test set of 2700 images, and both models retained 100% recall on an independent external set. The camera_type labels additionally support fine-grained classification and transfer-learning studies. Bounding boxes are not provided, so users targeting object detection must add their own localisation annotations before training detectors.
• Practitioners in digital forensics and law enforcement technology can use the data to develop and evaluate tools that automatically locate civilian cameras in imagery of crime scene surroundings, supporting faster canvassing for video evidence during the first hours of an investigation. It should be noted that this forensic use is the motivating application for the dataset rather than a property of the images themselves: the collection contains photographs of surveillance cameras, not photographs of crime scenes, and it is therefore equally applicable outside the forensic domain.
• Beyond forensics, the images can support research on privacy auditing, mapping of surveillance infrastructure in public space, urban studies, and smart-city applications, as well as educational use in computer vision and machine learning courses. The authors note that a resource which makes surveillance cameras easier to detect automatically has a dual-use character: the same capability that supports evidence canvassing and privacy auditing could in principle assist someone seeking to evade or disable cameras. The collection contains no location data and no imagery of specific protected sites, and the authors judge the benefit to public-interest research and oversight to outweigh that risk, but users are asked to consider the point when building on the dataset.

## 2 Background

Video footage recorded by civilian closed-circuit television (CCTV) systems is one of the most frequently used sources of digital evidence in criminal investigations [1]. When officers arrive at a crime scene, nearby cameras must be identified quickly, because many systems overwrite their recordings within days and the evidential value of footage is highest during the first hours of an investigation. In current practice this camera canvassing is performed manually: officers walk the perimeter of the scene, visually scan buildings and street furniture, and approach owners, a process that can take several hours. The dataset described in this article [2] was compiled to support the development of computer vision models that recognise surveillance cameras in imagery captured at or around a scene, so that camera locations can be identified automatically. The closest public resource, released by Sheng et al. [3], approaches the problem from the opposite direction: a camera detection model developed from 13,240 manually labelled street-view images, of which 861 contained a total of 977 cameras, was applied to 1.6 million street-view images across sixteen cities to estimate camera density. In that setting the devices appear only as small, distant objects within wide street scenes; no openly licensed collection of close-range, multi-viewpoint photographs of the devices themselves was found. The present dataset was assembled to fill that gap and served as the positive-class source material for camera-recognition experiments based on YOLOv8 [4] and ResNet-50 [5] classifiers.

## 3 Data Description

The dataset [2] is published on Zenodo (version doi:10.5281/zenodo.22669096; concept doi:10.5281/zenodo.19565334, which always resolves to the latest version) and comprises an image archive, combined_dataset.zip, a lightweight annotation file (labels.csv), a documented Python loading script (load_dataset.py), a CHECKSUMS.txt file listing SHA-256 checksums of the released files, and a README.md file. Extracting the archive yields one folder, combined_dataset/, containing 1421 image files named sequentially from camera_1 to camera_1421, without subfolders. All files are provided in JPEG (.jpg) format, in RGB colour space, standardised to a uniform resolution of 640 × 640 pixels. This uniformity was verified programmatically across the full archive: the minimum, maximum and modal dimensions of the 1421 files are all 640 × 640 pixels. Earlier versions of labels.csv carried per-image width_px and height_px columns; because every image has the same dimensions these columns held a single constant value on all rows and have been removed, the statement above replacing them. A check column present in earlier versions has likewise been removed: it was an internal annotator work-flag used to route ambiguous images to a second review, and that review is now complete, so the flag carries no meaning for downstream users.

The annotation file contains one row per image with the columns image_id, filename, context (in_situ for cameras photographed in real environments; product for manufacturer-style photographs), and camera_type, which records the form factor of the device depicted using the controlled vocabulary dome, bullet, ptz, box, consumer_ip, multi_sensor, other. Table 1 summarises the distribution of the context label, Table 2 that of the camera_type label, and Table 3 cross-tabulates the two. The collection covers the following camera categories:

• **Dome cameras** (camera_type = dome, 239 images, 16.8%) – indoor and outdoor dome-style cameras mounted on ceilings or walls;
• **PTZ (pan-tilt-zoom) cameras** (camera_type = ptz, 380 images, 26.7%) – steerable cameras on poles or buildings, including police and municipal installations;
• **Bullet cameras** (camera_type = bullet, 291 images, 20.5%) – elongated weatherproof cameras typically mounted outdoors on walls or poles;
• **Fixed box cameras** (camera_type = box, 186 images, 13.1%) – traditional rectangular cameras, often seen in industrial or transport settings;
• **Multi-sensor cameras** (camera_type = multi_sensor, 195 images, 13.7%) – dual- or multi-lens camera units, and mounts carrying several distinct camera heads, for wide-area coverage;
• **Consumer IP and doorbell cameras** (camera_type = consumer_ip, 97 images, 6.8%) – indoor Wi-Fi cameras with infrared illuminators and video doorbell units, representative of residential surveillance devices.
• **Other cameras** (camera_type = other, 33 images, 2.3%) – images in which a surveillance camera is present but does not correspond to any of the six form factors above, or in which the device is shown as a rendering or diagram rather than as a photograph of a physical unit.

**Table 1:** Class distribution of the context label in labels.csv.

Context	Number of images
in_situ (installed camera in a real environment)	718
product (manufacturer-style image)	703
Total	1421

**Table 2:** Class distribution of the camera type label in labels.csv.

Camera type	Number of images
Dome	239
PTZ (pan-tilt-zoom)	380
Bullet	291
Fixed box	186
Multi-sensor	195
Consumer IP and doorbell	97
Other	33
Total	1421

**Table 3:** Distribution of the camera type label cross-tabulated with the context label.

Camera type	in_situ	product	Total
Dome	93	146	239
PTZ (pan-tilt-zoom)	106	274	380
Bullet	154	137	291
Fixed box	162	24	186
Multi-sensor	169	26	195
Consumer IP and doorbell	16	81	97
Other	18	15	33
Total	718	703	1421

The images include both real-world in-situ photographs, showing cameras installed in public or private spaces, and manufacturer product images captured against plain or transparent backgrounds. Fig. 1 shows representative examples from the collection.

**Fig. 1:**
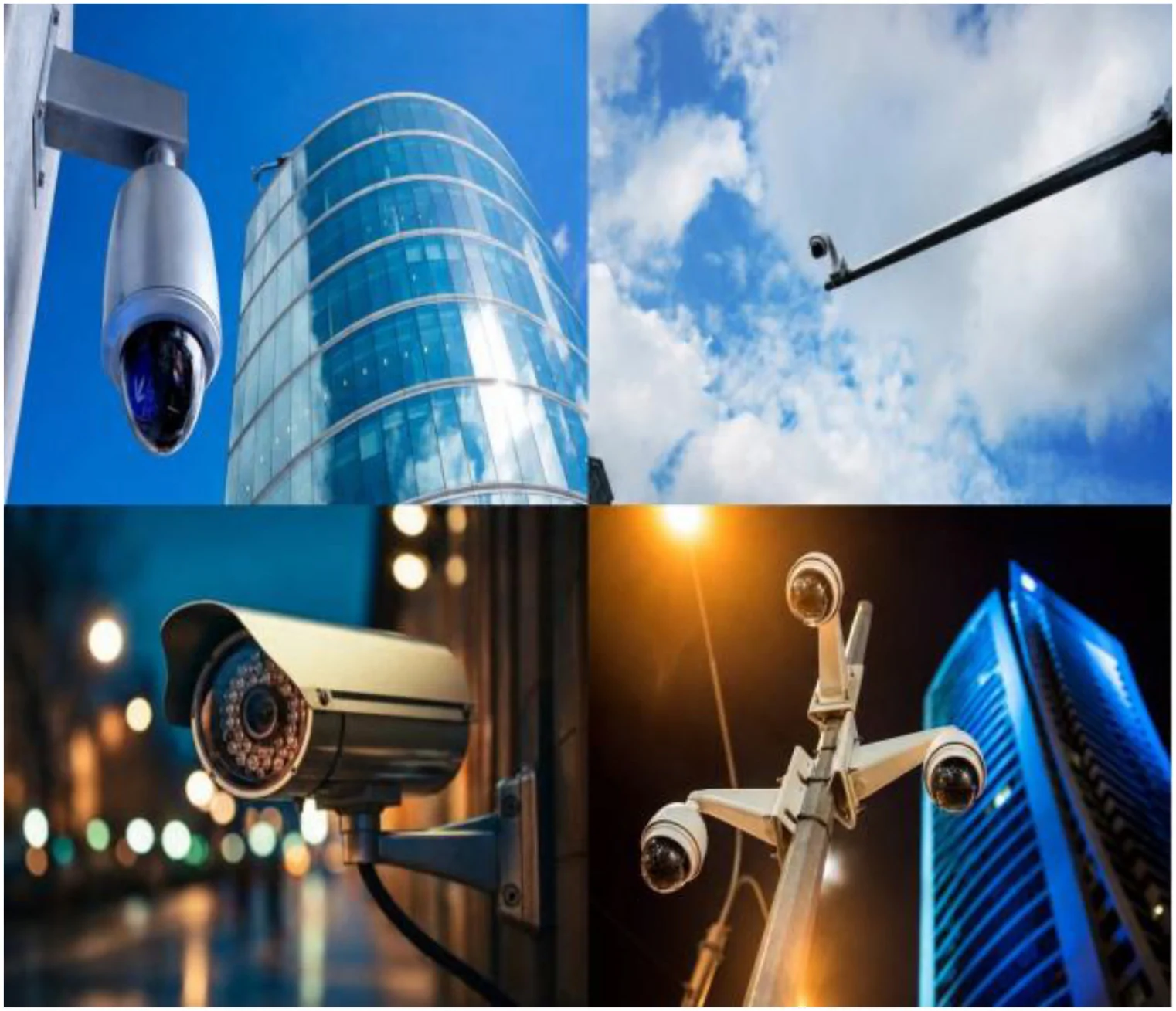
Example images from the dataset [2]: wall-mounted dome and bullet cameras photographed at close range (left column) and pole-mounted cameras in urban settings (right column).

## 4 Experimental Design, Materials and Methods

**Image collection -** Candidate images were located by open-source intelligence (OSINT) searching of publicly accessible web sources, open image repositories, and manufacturer product pages, using device terms (CCTV, security, surveillance, dome, bullet and PTZ cameras, video doorbells) combined with context terms (street, wall-mounted, pole, indoor, building facade) under licence filters restricting results to freely reusable material. Commercial stock-photography repositories were excluded. Each source was validated manually for its reuse terms before retrieval; images were then retrieved with custom Python scripts and validated manually a second time for licence status and content. Candidate images were selected to represent the full range of conditions under which surveillance cameras are encountered in practice, including different form factors, mounting heights and positions, viewing angles, indoor and outdoor environments, times of day, weather conditions, and backgrounds. Manufacturer product photographs were included alongside in-situ photographs so that the close-up appearance of camera housings is also represented. Per-image source URLs were not retained in this release; adding them is the primary planned extension, as noted in the Limitations section.

**Curation and standardisation -** Every candidate image was reviewed manually by the authors and through automated quality checks implemented as custom Python scripts using Pillow and NumPy. The checks verified file integrity, computed a perceptual hash to detect exact and near-duplicate files, rejected images below a minimum usable resolution, and flagged severe blur or extreme exposure; flagged files were adjudicated manually rather than deleted automatically. An image was retained only if at least one surveillance camera was clearly visible at adequate quality; duplicates, ambiguous items, and low-quality files were excluded. Retained files were renamed sequentially (camera_<ID>, 1 to 1421), consolidated into a single flat directory, converted to JPEG (RGB), and standardised to 640 × 640 pixels. Resizing scaled each image directly to 640 × 640 without preserving the original aspect ratio, so non-square source images are horizontally or vertically stretched; no cropping or padding was applied, and the full framing of every source image is therefore retained. No augmentation was applied to the released images; augmentation was used only to build the training split of the validation experiments described below.

**Annotation -** Each image was assigned two labels by visual inspection: the context label, distinguishing in-situ photographs from manufacturer-style product images, and the camera_type label, recording the form factor using the seven-value vocabulary defined in the README. Both labels were assigned independently by two annotators (D. Rangelov and N. Rangelov) working from written category definitions, with rules agreed in advance for recurring borderline cases: a plain or synthetic marketing background makes an image product, whereas any identifiable architectural, street or interior context makes it in_situ; a single mount carrying two or more camera heads is multi_sensor; and a dome-shaped unit on a pendant or pole mount with a motorised drum base is ptz rather than dome. Disagreements were resolved by joint review, so the released file contains no unresolved labels. The internal work-flag used during that review (the check column of earlier versions) has been removed.

**Technical validation -** To assess the suitability of the collection for machine learning applications, the images were used as positive-class source material in binary camera/no-camera image classification experiments. Negative-class images were drawn from public datasets, including Microsoft COCO [6] and Open Images [7]. The pooled source material was partitioned into training, validation and test subsets in a 70/20/10 ratio before any augmentation was applied, and augmentation was then applied independently within each subset, so that no augmented copy of an image can appear in more than one subset. The augmentation operations were horizontal and vertical flips, 90-degree rotations, rotations of up to ±15 degrees, brightness variation of ±25%, exposure variation of ±15%, saturation variation of ±30%, and additive noise of up to 2%. The composition of the held-out test set is given in Table 4: it contains 2700 images, but these derive from 505 independent source photographs, an average of 5.4 augmented variants of each. Augmented variants of the same source image are highly correlated, so the effective number of independent observations behind the metrics reported below is 505 rather than 2700, and the figures should be read accordingly. The 236 camera-positive source images are all part of the dataset described in this article, and the 269 camera-negative source images were drawn from COCO and Open Images. Both subsets are listed image by image in test_images.csv and test_images_negative.csv in the deposit [2].

**Table 4:** Composition of the held-out test set used in the technical validation.

Test-set component	Unique source images	Images after augmentation
Camera (positive)	236	1350
No camera (negative)	269	1350
Total	505	2700

Two pipelines were trained on this material, both using transfer learning: a YOLOv8-m classification model [4] (input size 640 × 640 px, batch size 16, SGD, initial learning rate 0.01, momentum 0.937, weight decay 0.0005, cosine schedule), fine-tuned from COCO-pretrained weights on an NVIDIA GeForce RTX 4060 GPU, and a ResNet-50-based classifier [5] (input size 224 × 224 px), fine-tuned from ImageNet-pretrained weights on the Roboflow platform. On the held-out test set the ResNet-50-based classifier reached 98.1% accuracy (precision 97.5%, recall 98.7%, F1 score 98.1%), and the YOLOv8-m model reached 82.0% accuracy (precision 84.9%, recall 82.0%, F1 score 81.6%).

The gap between the two figures reflects the training pipelines rather than the data: the models operate at different input resolutions (224 × 224 against 640 × 640), the managed ResNet-50 pipeline fitted its in-distribution partition tightly at 99.2% validation accuracy, and YOLOv8-m adopted a recall-oriented operating point. Both models were fine-tuned from pretrained weights, so neither was disadvantaged by initialisation. The gap does not persist out of distribution. Both models were additionally evaluated on an external set of 100 images, 19 camera-positive and 81 camera-negative, collected independently of the material used for training and left unaugmented, so that every image in it is a distinct photograph. On that set the two are statistically indistinguishable (YOLOv8-m 84.0%, 95% confidence interval 75.6–89.9; ResNet-50 83.0%, 95% CI 74.5–89.1), both achieving 100% recall on the 19 camera-positive images, with every error falling on the negatives. These figures characterise the suitability of the dataset for machine learning use; the models are not a contribution of this data article.

## Limitations

The release contains only positive-class images (every image depicts at least one surveillance camera). Negative-class material must be sourced separately. Annotation is limited to two per-image labels, context (in-situ or product) and camera_type (form factor); no bounding boxes or segmentation masks are provided, so users targeting object detection or localisation must add their own annotations. Images were located through searches restricted to freely reusable material, and the reuse terms of every candidate source were checked manually before retrieval, but per-image source URLs were not retained. An individual image therefore cannot be traced back to its origin, and a user wishing to credit the original creator of a specific image cannot do so from the deposit alone. The CC BY 4.0 licence applied to the deposit covers the curated collection, that is the selection, standardisation, annotation, documentation and validation contributed by the authors; it is not a re-licensing of any underlying image. Recovering per-image provenance and licence metadata is the primary planned extension of this dataset, and any image whose upstream terms prove incompatible with redistribution will be removed on request. Standardisation stretched non-square source images to 640 × 640 pixels, so apparent housing proportions differ from the originals; users to whom exact geometry matters should account for this. Class balance reflects what is available under open licences on the web rather than the true deployed population of surveillance devices, and the imbalance is uneven across the two label dimensions: product imagery skews towards PTZ (274 of 380) and consumer IP units (81 of 97), whereas in-situ imagery skews towards fixed box (162 of 186) and multi-sensor installations (169 of 195). Models trained on the collection without accounting for this may learn background cues alongside device appearance. Although all images are standardised to 640 × 640 pixels, visual quality varies across sources, and a substantial portion are manufacturer product photographs against plain backgrounds, whose visual appearance differs from cameras installed in real environments. Geographic coverage and manufacturer distribution were not controlled during collection, so regional and brand biases may be present, and near-duplicate views of the same camera model may occur. Night-time, adverse-weather, occluded and low-resolution conditions are represented but not systematically sampled. A reproducibility limitation applies to the technical validation rather than to the data: the ResNet-50 classifier was trained through a managed platform whose low-level hyperparameters, including the optimiser, learning-rate schedule and weight decay, are set by the platform and are not exposed to the user, so that pipeline cannot be reproduced exactly from the information available to the authors. Future releases are intended to address these points by adding per-image source and licence metadata, bounding-box annotations, and additional images covering the under-represented form factors and conditions; the CC BY 4.0 licence permits the community to extend the annotations independently.

## Ethics Statement

The authors have read and follow the ethical requirements for publication in Data in Brief and confirm that the current work does not involve human subjects, animal experiments, or any data collected from social media platforms. The images depict devices rather than people, and no systematic screening for incidentally captured personal data was carried out during curation; every image was, however, reviewed manually twice for content. Human figures appear predominantly within manufacturer marketing imagery, in which models or installers are posed with the product, and where people appear in real-world scenes they are incidental, distant, and never the subject of the photograph. A post-hoc automated face-detection pass over the full collection found no prominent identifiable faces outside such product imagery. No image carries location metadata, and no image was selected to depict a specific individual, vehicle or address. The authors will remove any image on request from a person who considers themselves identifiable in it, or from a rights holder.

## Credit Author Statement

**Dimitar Rangelov:** Conceptualization, Methodology, Data curation, Supervision, Writing – original draft; **Nikolay Rangelov:** Investigation, Data curation, Writing – original draft; **Radoslav Miltchev:** Resources, Validation, Writing – review & editing; **Evgeni Genchev:** Data curation, Validation, Writing – review & editing.

## Data Availability

ZenodoSurveillance Camera Image Dataset for Visual Classification in Criminal Investigation Research (CCTV Cameras) (Original data)
ZenodoSurveillance Camera Image Dataset for Visual Classification in Criminal Investigation Research (CCTV Cameras) (Original data)

## References

[1] Ashby M.P.J. (2017). The value of CCTV surveillance cameras as an investigative tool: an empirical analysis. Eur. J. Crim. Polic. Res..

[2] D. Rangelov, N. Rangelov, R. Miltchev, E. Genchev, Surveillance Camera Image Dataset For Visual Classification in Criminal Investigation Research (CCTV Cameras), Zenodo, v6, 2026. 10.5281/zenodo.22669096. Versions v4 and v5 revised the deposit documentation only; the concept DOI https://doi.org/10.5281/zenodo.19565334 always resolves to the latest version.

[3] Sheng H., Yao K., Goel S. (2021). Proceedings of the 2021 AAAI/ACM Conference on AI, Ethics, and Society (AIES ’21).

[4] G. Jocher, A. Chaurasia, J. Qiu, Ultralytics YOLOv8 [software], Ultralytics, 2023. https://github.com/ultralytics/ultralytics (accessed 8 July 2026).

[5] He K., Zhang X., Ren S., Sun J. (2016). Proceedings of the IEEE Conference on Computer Vision and Pattern Recognition (CVPR).

[6] Lin T.-Y., Maire M., Belongie S., Hays J., Perona P., Ramanan D., Dollár P., Zitnick C.L., Fleet D., Pajdla T., Schiele B., Tuytelaars T. (2014). Computer Vision – ECCV 2014, Lecture Notes in Computer Science.

[7] Kuznetsova A., Rom H., Alldrin N., Uijlings J., Krasin I., Pont-Tuset J., Kamali S., Popov S., Malloci M., Kolesnikov A., Duerig T., Ferrari V. (2020). The open images dataset V4: unified image classification, object detection, and visual relationship detection at scale. Int. J. Comput. Vis..

